# Supplementary material for: Cytokines Stimulated by IL-33 in Human Skin Mast Cells: Involvement of NF-κB and p38 at Distinct Levels and Potent Co-Operation with FcεRI and MRGPRX2
Source: Int J Mol Sci. 2021 Mar 30;22(7):3580. doi: 10.3390/ijms22073580 (PMC8036466; doi:10.3390/ijms22073580)
Supplement: Supplementary file 1 [file ijms-22-03580-s001.zip › Supplementary files/Supplementary figures S1 and S2 and S3_revised.docx]

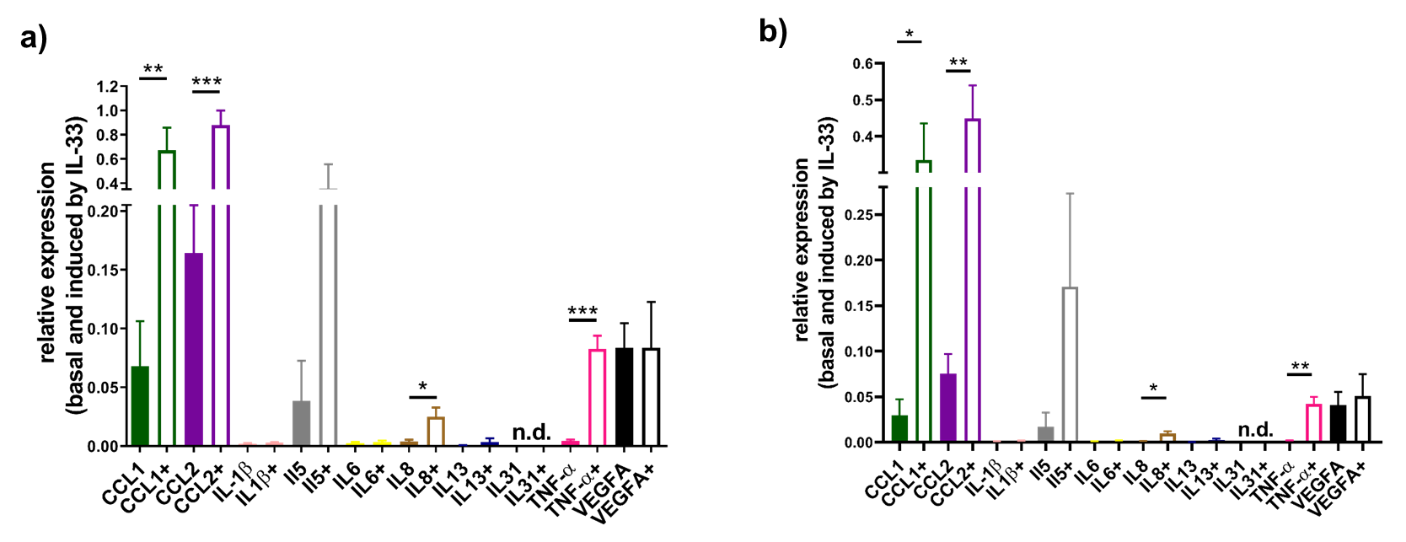


**Supplementary figure 1: Levels of cytokine transcripts in skin MCs at baseline and following IL-33 stimulation.** Skin-derived MCs (at 5 x 10^5^ cells/mL) in minimal medium were stimulated for 90 minutes with IL-33 or kept without stimulus for baseline expression. Cytokines were quantified by RT-qPCR. Basal (plain cytokine name) and IL-33-induced (cytokine name followed by “+”) expression levels of cytokines relative to **(a)** Cyclophilin B and **(b)** GAPDH. The data are presented as mean of 7 experiments ± SEM. n. d. = not detected for any condition. The datasets are the same as in Figure 1a and 1b, only normalized differently. *p<0.05, **p<0.01, ***p<0.001


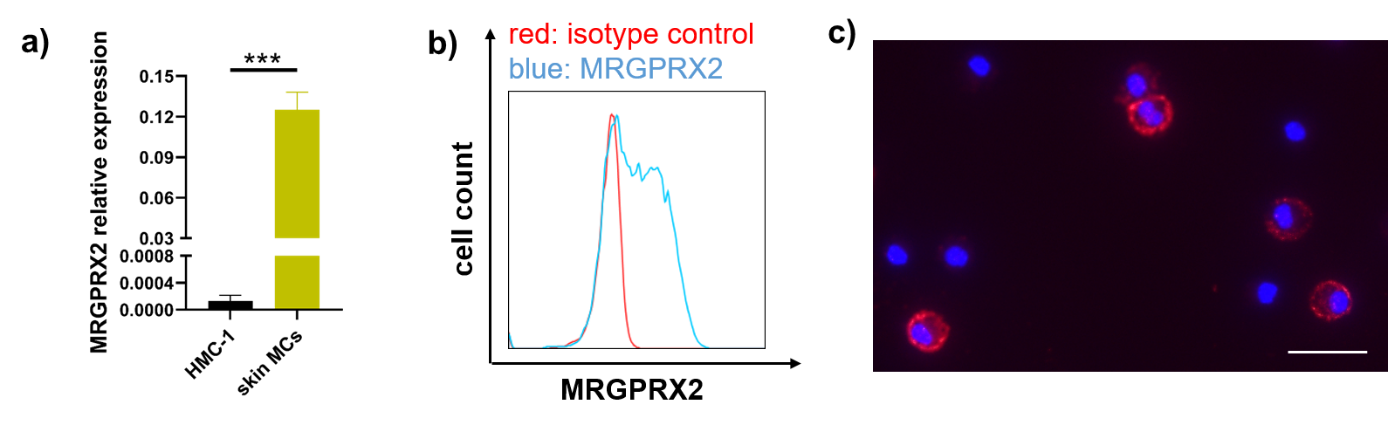


**Supplementary figure 2: MRGPRX2 is expressed in primary skin MCs.** Cultured **s**kin-derived MCs were analyzed regarding MRGPRX2 expression on mRNA and protein level. **(a)** mRNA of MRGPRX2 in MCs in comparison to HMC-1 cells (reportedly expressing MRGPRX2 at low level [1]), normalized to GAPDH. The data is presented as mean of 7-10 experiments (independent MC cultures in case of skin MCs) ± SEM. ***p<0.001. **(b)** representative FACS analysis (cell surface staining of MRGPRX2) and **(c)** representative fluorescence microscopy photograph. Scale bar 20 µm.

1. Hermans, M.A.W.; Stigt, A.C.v.; Meerendonk, S.v.d.; Schrijver, B.; Daele, P.L.A.v.; Hagen, P.M.v.; Splunter, M.v.; Dik, W.A. Human mast cell line HMC1 Expresses functional Mas-related G-protein coupled receptor 2. *Front Immunol* **2021**, 10.3389/fimmu.2021.625284, doi:10.3389/fimmu.2021.625284.


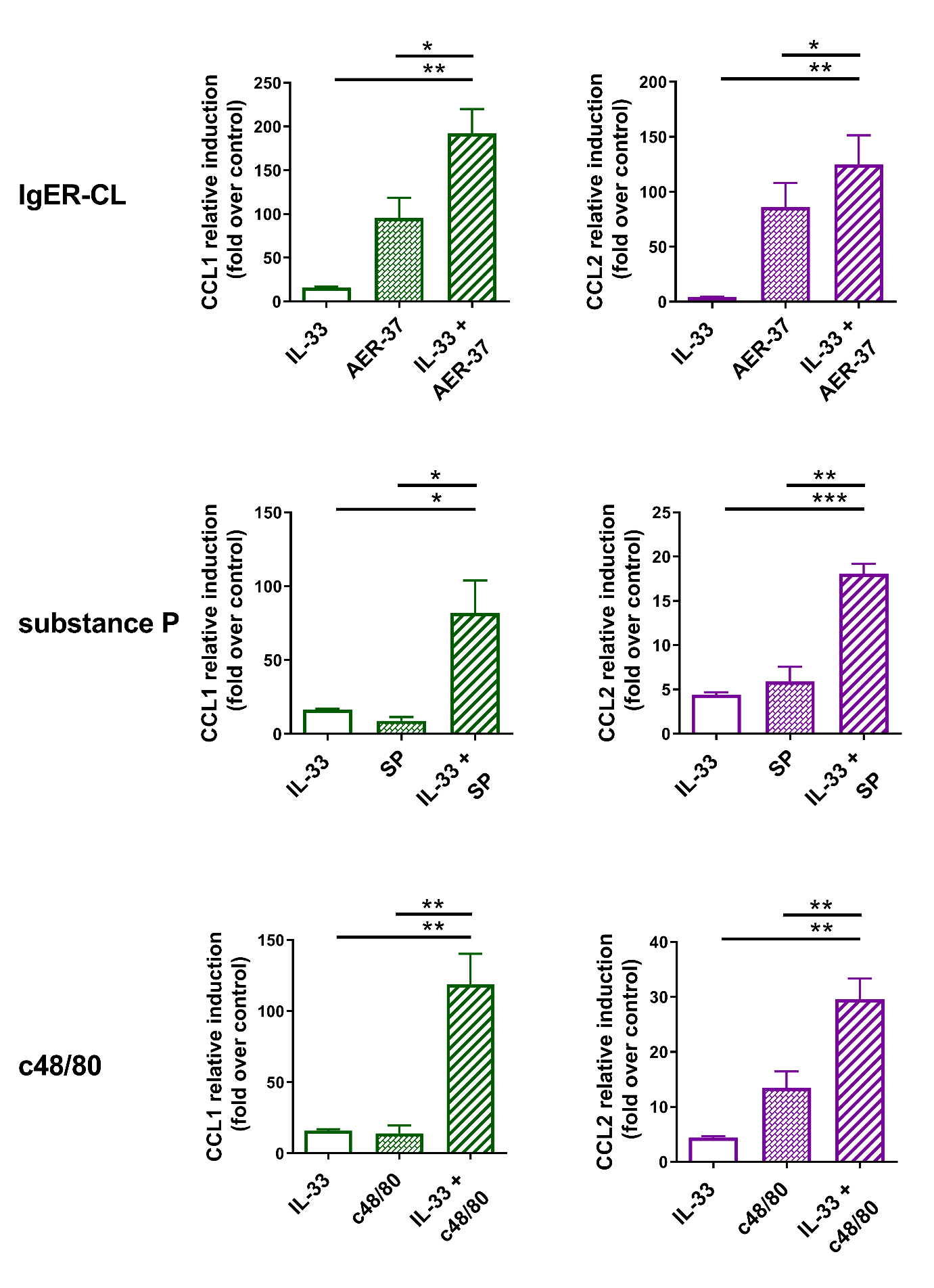


**Supplementary figure 3: CCL1 and CCL2 chemokine transcripts elicited by the combined activation of IL-33 plus FcεRI-CL or plus MRGPRX2 agonists.** Cells were stimulated with IL-33 or AER-37 (FcεRI-CL) or SP or c48/80 (MRGPRX2-ligands) for 105 minutes or pre-treated with IL-33 for 15 minutes prior to the secondary stimuli for 90 minutes, exactly as in Figure 5a. CCL1 and CCL2 were quantified by RT-qPCR. The data are presented as mean of 5-7 experiments ± SEM. * p<0.05, ** p<0.01, ***p<0.001
