# Supplementary material for: Cytokines Stimulated by IL-33 in Human Skin Mast Cells: Involvement of NF-κB and p38 at Distinct Levels and Potent Co-Operation with FcεRI and MRGPRX2
Source: Int J Mol Sci. 2021 Mar 30;22(7):3580. doi: 10.3390/ijms22073580 (PMC8036466; doi:10.3390/ijms22073580)
Supplement: Supplementary file 1 [file ijms-22-03580-s001.zip › Supplementary files/Supplementary Material and Methods.docx]

**Supplementary Material and Methods**

**Flow cytometry**

MCs were blocked for 15 min at 4 °C with human AB-serum (Biotest, Dreieich, Germany) and incubated with antibodies for 30 min at 4 °C [1]. Anti-human MRGPRX2 (clone K125H4, Biolegend San Diego, CA, USA) was used at 0.15 µg/mL and PE-labelled mouse IgG2b-PE (clone eBMG2b, eBioscience, San Diego, CA, USA) served as isotype control. MRGPRX2 surface expression was measured on the MACSQuant (Miltenyi-Biotec, Bergisch Gladbach, Germany). Data were analyzed using the FlowJo analysis software (FlowJo LLC, Ashland, OR, USA).

**Immunofluorescence**

Suspensions of stained skin MCs (anti-human MRGPRX2 antibody, clone K125H4, Biolegend) were washed once with ice cold 1x PBS, fixed with 4% paraformaldehyde and then resuspended in 110 µl PBS for cytospin preparation. Approximately 50.000 cells were spun on a glass slide for 3 minutes at 500 rpm and covered with DAPI containing Fluoromount-G^TM^ (Invitrogen). Photographs were taken on the BZ-X810 Keyence microscope (objective 40x) [2].

**Cell line**

The leukemic MC line HMC-1 was kindly provided by Dr. J.H Butterfield [3]. Cells were cultured at 5x10^5^-1x10^6^/mL in the same medium as used for skin MCs and cells were fed three times a week.

**MRGPRX2 RT-qPCR**

RT-qPCR for MRGPRX2 was performed as described in the main manuscript using the following primers, as described [2]:5’ –GGATCAGGAAGACCGGGATCA-3’ and 5’–CGGCCTGGGGAACAGAAAGT-3’.

**References**

1. Wang, Z.; Guhl, S.; Franke, K.; Artuc, M.; Zuberbier, T.; Babina, M. IL-33 and MRGPRX2-Triggered Activation of Human Skin Mast Cells-Elimination of Receptor Expression on Chronic Exposure, but Reinforced Degranulation on Acute Priming. *Cells* **2019**, *8*, doi:10.3390/cells8040341.

2. Babina, M.; Wang, Z.; Roy, S.; Guhl, S.; Franke, K.; Artuc, M.; Ali, H.; Zuberbier, T. MRGPRX2 Is the Codeine Receptor of Human Skin Mast Cells: Desensitization through beta-Arrestin and Lack of Correlation with the FcepsilonRI Pathway. *J Invest Dermatol* **2020**, 10.1016/j.jid.2020.09.017, doi:10.1016/j.jid.2020.09.017.

3. Butterfield, J.H.; Weiler, D.; Dewald, G.; Gleich, G.J. Establishment of an immature mast cell line from a patient with mast cell leukemia. *Leuk Res* **1988**, *12*, 345-355, doi:10.1016/0145-2126(88)90050-1.
